# A mathematical model to predict mean time to delivery following cervical ripening with dinoprostone vaginal insert

**DOI:** 10.1038/s41598-019-46101-2

**Published:** 2019-07-09

**Authors:** Fanny Levast, Guillaume Legendre, Hady El Hachem, Patrick Saulnier, Philippe Descamps, Philippe Gillard, Pierre-Emmanuel Bouet

**Affiliations:** 1 Department of Obstetrics and Gynecology, Angers University Hospital, Angers, France; 2 Department of Reproductive Medicine, Clemenceau Medical Center, Beirut, Lebanon; 3 Department of Methodology and Biostatistics, Angers University Hospital, Angers, France

**Keywords:** Pregnancy outcome, Outcomes research

## Abstract

The main objective of our study was to analyze the mean time to delivery following cervical ripening with a 10 mg dinoprostone vaginal insert. We performed a retrospective observational study at the level III maternity ward of Angers university hospital. We included all women who had cervical ripening with dinoprostone between January 1^st^, 2015 and September 30^th^, 2016. Overall, 405 patients were included, and 59.3% (240/405) were nulliparous. The mean time to delivery was 20h39 min ± 10h49 min. 21% of deliveries (86/405) occurred between midnight and 6 h a.m., and the cesarean section rate was 33% (132/405). Multiple regression analysis showed that nulliparity, overweight (BMI ≥ 25), a closed cervix on initial examination and the absence of premature rupture of membranes (PRM) all significantly increased the mean time to delivery. We developed a mathematical model integrating the aforementioned factors and their impact to help predict the mean time to delivery following cervical ripening with dinoprostone vaginal insert: Y = 961.188–80.346 × parity + 21.437 × BMI–165.263 × cervical dilation–241.759 × PRM. This equation allows obstetricians to calculate a personalized time to delivery for each patient, allowing a precise scheduling of dinoprostone insert placement, and thus improving the organization in busy maternity wards.

## Introduction

Induction of labor (IOL) is nowadays a common obstetrical intervention on maternity wards worldwide, with an estimated yearly rate of 20% in France^1,2^. The ultimate goal of IOL is to artificially provoke uterine contractions, before their natural occurrence, thus leading to labor and delivery. A successful IOL requires the cervix to be mature and ready for vaginal delivery, which is usually assessed by the Bishop score. The Bishop score was originally described to assess the likelihood of vaginal delivery in multiparous women but is nowadays used for assessment of women considered for IOL. A Bishop score >6 is usually considered adequate for IOL^3^, whereas patients with scores ≤6 require cervical ripening before IOL. Many cervical ripening methods are currently available, such as misoprostol, Foley catheters, dinoprostone pessaries and intra-vaginal dinoprostone^4–7^. There is a wide difference in the use of these methods across maternities and countries. In France, the most commonly used method for cervical ripening is intravaginal dinoprostone and more specifically the dinoprostone vaginal insert^1^.

Very few studies have assessed the mean time to delivery (TTD) following cervical ripening with dinoprostone vaginal insert. Wing *et al*. reported a mean TTD of 32.8 hours after placement of dinoprostone vaginal insert, with 43.1 hours recorded for nulliparous and 20.1 hours for multiparous women^8^. Namaky *et al*.^9^ reported a mean TTD of 14.9 hours when cervical ripening (with vaginal dinoprostone or misoprostol) was performed in the morning, compared to 21.4 hours when performed in the afternoon, regardless of parity and delivery method^9^. Overall, the reported times in the few available studies are quite variable and heterogeneous, making counseling more difficult for women before IOL. Indeed, studies have shown lower satisfaction rates and more unrealistic expectations in women undergoing IOL compared to spontaneous labor^10^. Moreover, it is important for obstetricians and midwives to be able to project the time of delivery, for organization and safety purposes, especially in busy maternity wards. Indeed, recent studies have reported increased maternal and fetal morbidity and mortality when deliveries occur at night^11–14^. Furthermore, some deliveries are considered high risk and require the intervention and coordination of several medical and surgical teams, such as cases of placenta praevia/accreta at high risk of postpartum bleeding, or cases requiring immediate neonatal surgery. Such deliveries are best scheduled to occur during the day, when the hospital is fully staffed, and all busy level III maternity wards have now developed organizational charts, in order to be able to plan and handle these complex cases. Having an adequate prediction tool for the delivery time following cervical ripening and IOL would certainly improve these organizational diagrams, by allowing a precise scheduling of insert placements. Unfortunately, the few studies reporting on the most appropriate timing of dinoprostone vaginal insert placement, based on Bishop score and parity, have yielded conflicting results^9,15–17^.

The main objective of our study was to assess the mean time to delivery following cervical ripening with a 10-mg dinoprostone vaginal insert. Our secondary end points were to determine the factors influencing the time to delivery, and to develop a mathematical model that would allow us to calculate the TTD for each woman, based on these factors.

## Methods

### Patients

We performed an observational retrospective study at the level III maternity ward of the Angers University Hospital. We included all patients receiving a 10-mg dinoprostone vaginal insert for cervical ripening between January 1^st^, 2015 and September 30^th^, 2016. We included all patients, regardless of age, gestation, parity, body mass index (BMI), and indication for cervical ripening. We excluded all patients who had double cervical ripening (dinoprostone with a mechanical method or two doses of dinoprostone), as well as patients with a contraindication to dinoprostone: twin pregnancies, dystocic presentations diagnosed before cervical ripening, scarred uteri, and allergies to prostaglandins E_2_. Patients with a previous history of cervical conization, cervical incompetence or cerclage were not excluded from the study, since these findings do not constitute a contraindication for cervical ripening with dinoprostone according to our departments’ protocols. All patients’ files were identified from the Angers University Hospital Medical Birth Registry.

This study was carried out in accordance with relevant guidelines and regulations. Our protocol was approved by the Ethics Committee of Angers University Hospital (Ref. 2017/70, dated October 25, 2017). Informed consent was obtained from all subjects or, if subjects are under 18 from a parent and/or legal guardian.

### Procedure and definitions

All patients had a 10-mg dinoprostone vaginal insert, which is commercialized in France under the name Propess® (Ferring Pharmaceuticals). It is worth noting that in France cervical ripening with prostaglandins can only be performed using dinoprostone insert (Propess®, Ferring Pharmaceuticals) or gel (Prostine®, Pfizer). At our hospital, we do not use dinoprostone gel (Prostine®). Misoprostol (Cytotec®, Pfizer) is no longer used for labor induction in France and is no longer available in the French market since March 2018, following many cases of obstetrical and neonatal complications that have led to several lawsuits^18^.

The decision of cervical ripening was made by the attending obstetrician during the morning round following the daily morning meeting. The Bishop score was calculated following a pelvic exam performed by the obstetrics and gynecology resident on the maternity ward. The Bishop score assesses the following: Cervical dilation, effacement, consistency and position, as well as fetal station, and gives points (0, 1 or 2) to each of the criteria. Patients with a score ≤6 were candidates for cervical ripening with dinoprostone, including patients who had PRM without spontaneous labor in the following 24 hours. The midwife on the maternity ward or on the high-risk pregnancy floor placed the insert in the posterior vaginal fornix. The patient stayed in the supine position for 30 minutes following placement, and a 2-hour fetal heart rate (FHR) monitoring was performed. FHR monitoring was repeated every 8 hours, or in case of start of labor or the occurrence of any complication. The insert was left in place until the start of labor, or for a maximum of 24 hours. Pelvic examinations were not systematic, and were only performed when active labor started or in cases of complication. In cases of uterine hypertonus or hyperkinesia leading to fetal heart rate anomalies, the insert was removed and an utero-relaxant agent (Nitronal, 1 mg) was given if needed. Labor was induced with oxytocin perfusion and with amniotomy as soon as possible if there was no premature rupture membrane. Start of labor was defined as the time when 3 or 4 uterine contractions were recorded over a 10-minute period along with cervical changes, or >4 cm cervical dilation regardless of the frequency of contractions^8,19^. At our center, we define labor induction failure as stagnation of the latent phase of labor for 6 to 9 hours from the onset of oxytocin perfusion or amniotomy.

### Outcome measures and definitions

We collected all relevant maternal and fetal characteristics, as well as data on the indications and modalities of cervical ripening, the labor progress, the delivery and the post-partum period. The mean time to delivery (TTD) was calculated from the time of the vaginal insert placement to the time of birth. We defined post-term pregnancy as pregnancy progressing beyond 41 weeks gestational age (GA)^20^, while we defined premature rupture of membranes (PRM) as rupture of the membranes before the onset of labor, regardless of gestational age^21^.

### Statistical analysis

Descriptive statistics were performed using Microsoft Excel 2016 and GraphPad Prism version 7.00 for Windows (GraphPad Software, La Jolla California USA, www.GraphPad.com). Univariate analysis of mean TTD was performed using Student’s t test and ANOVA. We also analyzed the cumulative delivery rate in patients who had cervical ripening with Propess® according to different maternal and obstetrical characteristics, and we performed survival analysis using Kaplan-Meyer estimates and a log-rank test to compare outcomes. A p-value < 0.05 was considered statistically significant. Moreover, we performed a multivariate linear regression analysis of the factors associated with TTD using SPSS version 15.0 for Windows (SPSS Inc., Chicago Illinois USA). The categorical variable “premature rupture of membranes (PRM)” was coded as 0 when membranes were intact at the start of labor and 1 when rupture was confirmed before. For the variable “parity”, nulliparity was chosen as the reference value and coded 0. Finally, for “cervical dilation”, “closed” was chosen as the reference value and coded 0, whereas “one finger” was code as 1 and “two fingers” was coded as 2.

## Results

### Clinical and demographic characteristics

**Figure 1:**
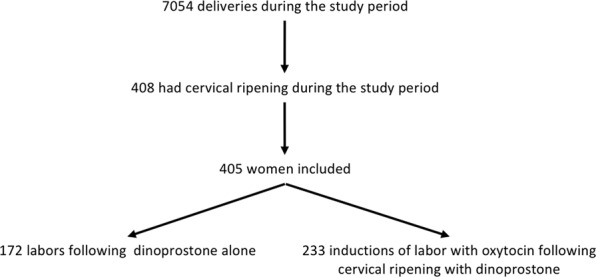
Study flowchart.

We included 405 patients (Fig. 1). Mean maternal age was 29 ± 5,7 years and 59.3% were nulliparous (Table 1). None of the patients had a previous history of cervical incompetence or cervical cerclage, whether in the current or previous pregnancies. 3 patients had a positive history of cervical conization. The gestational age at the time of insertion varied from 34^+0^ to 42^+0^ weeks, with 2% (8/405) occurring before 35 weeks GA and 28.6% (116/405) occurring after 41 weeks GA. A quarter of patients (24.9%) (101/405) had cervical ripening for post-term pregnancy while 15% (60/405) had PRM, preeclampsia, intra uterine growth restriction (IUGR) and decreased active fetal movements (AFM) accounted for 11% of indications of cervical ripening (Table 2). The other indications, in descending order, were: gestational diabetes, cholestasis of pregnancy, and fetal macrosomia. The mean Bishop score before insertion was 3.75% (303/405) of inserts were placed between 9 h and 12 h a.m., 16% (66/405) between 12 h and 15 h p.m., 2% (9/405) between 15 h and 18 h p.m., 3.5% (14/405) between 18 h p.m. and midnight, and 3.5% (13/405) between midnight and 6 h a.m. 42% of patients went into spontaneous labor following insertion (Table 2). The overall cesarean section rate was 33% and the vaginal delivery rate was 67% (Table 2). In women who had a cesarean section, approximately 80% were nulliparous, 68% had a Bishop score ≤3 and 56% were not in labor. The major indications for cesarean section following placement of the dinoprostone vaginal insert were: fetal distress (59.1%), failed induction of labor (18.2%), and failure to progress (12.1%) (Table 2).

**Table 1:** Maternal and neonatal characteristics.

	Dinoprostone vaginal insertN = 405
Age (years)	29,3 ± 5,7
≤25 (%)	103 (25.4)
26–35	246 (60.8)
>35	56 (13.8)
Gestation	2,3 ± 1,2
Parity	0,7 ± 1,1
Nulliparous (%)	240 (59.3)
BMI (kg/m2)	25,8 ± 6,5
<25 (%)	211 (52.1)
≥25	187 (46.2)
Non-available	7 (1.7)
Term (GA)	38,8 ± 2,5
Fetal Weight (grams)	3149,5 ± 607,8
<2500	69 (17)
>3900	39 (6, 9)
Neonatal pH	7,22 ± 0,09
APGAR score<7	
1 min	48 (8,11)
3 min	16 (4,0)
5 min	2 (0,5)
Hospitalization	
Maternity	364 (89.9)
Mother/baby Unit	22 (5.4)
Neonatal intensive care	19 (4.7)

**Table 2:** Obstetrical characteristics and outcome

	Dinoprostone vaginal insert N = 405
Indications of cervical ripening	
Post-term pregnancy	101 (24.9)
Premature rupture of membranes	60 (14.8)
Intra Uterine Growth Restriction	46 (11.4)
Decreased active fetal movements	44 (10.9)
Pre-eclampsia	43 (10.6)
Fetal macrosomia	34 (8.4)
Cholestasis of pregnancy	32 (7.9)
Gestational diabetes	31 (7.6)
Non-medical reason	12 (3)
Other	2 (0.5)
BISHOP score	3 ± 1,4
0	18 (4.4)
1	61 (15.1)
2	63 (15.6)
3	103 (25.4)
4	98 (24.2)
5	57 (14.1)
≥$6$	5 (1.2)
Active labor following dinoprostone insertion	172 (42.5)
Premature rupture of membranes	128 (31.6)
Delivery route	
Vaginal delivery	230 (56.8)
Operative vaginal delivery	43 (10.6)
Cesarean section	132 (32.6)
Cesarean section Indications	
Fetal distress	78 (59.1)
Failed induction of labor	24 (18.2)
Stagnation de la dilatation	16 (12.1)
Non-engagement of fetal head	9 (6.8)
Other	5 (3.8)

Data expressed as mean ± standard deviation or n (%).

### Mean time to delivery and influencing factors

**Figure 2:**
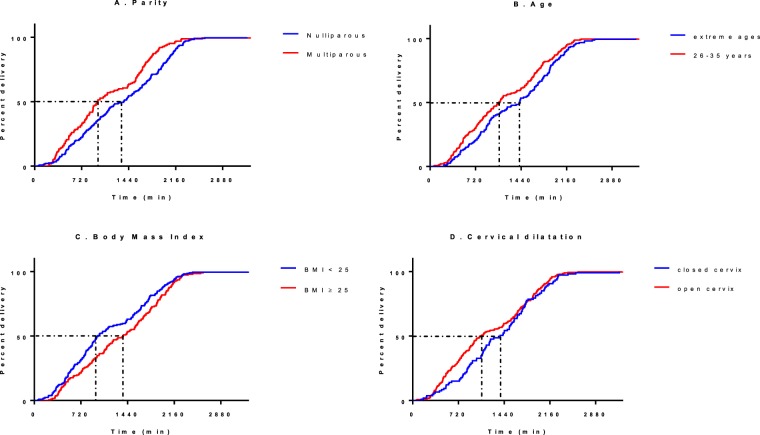
Kaplan-Meyer survival curves. (**A**) according to parity, *p* = *0.0003*. (**B**) according to age, *p* = *0.0104*. (**C**) according to BMI, *p* = *0.0254*. (**D**) according to initial cervical dilation*, p* = *0.1099*.

All patients who had cervical ripening with dinoprostone vaginal insert had delivered after 40 hours. The mean TTD was 20h39 min ± 10h49 min, with a median of 19h15 min (interquartile interval [11h40 min–29h37 min]) (Table 3). 21% of deliveries occurred between midnight and 6 h a.m. 173 patients went into labour with the dinoprostone insert in place less than 24 hours after its insertion. In this subgroup of patients, the mean time to start of labour was 9h32 min ± 3h15 min. The mean time to delivery was significantly longer in: nulliparous women (22h05 min versus 18h36 min, p = 0.001), age ≤25 or ≥36 years (22h07 min in ≤25 years, versus 18h35min in 26–30 years, 20 h46min in 31–35 years, 22h52min in ≥36, p = 0.02), BMI ≥ 25 (19h12 min in <25, versus 21h35 min in 25–29, 22h48 min in ≥30, p = 0.02), “closed” cervix (22h30 min versus 20h, p = 0.04), and absence of PRM (21h58 min versus 17h48 min, p < 0.001). There was no difference in the mean TTD between women with Bishop score ≤3 compared to >3 (21h24 min versus 19h31 min, p = 0.09), no difference according to the indication of cervical ripening (p = 0.42) and no difference according to gestational age (<37 weeks compared to ≥37 weeks: 21h19 min versus 20h37 min, p = 0,73). On the other hand, there was a statistically significant difference in the cumulative delivery rate over time according to parity, age, and BMI (p < 0.05), but not according to initial cervical dilation (Fig. 2). The multiple linear regression analysis showed that the following factors significantly affected the time to delivery following placement of the dinoprostone vaginal insert: “parity”, “BMI”, “cervical dilation”, and “PRM”. Age was not significantly associated with TTD (Table 4). Therefore, the regression equation was: “Y = 961.188–80.346 × *parity* + 21.437 × *BMI*–165.263 × *cervical dilation*–241.759 × *PRM*”.

**Table 3:** Mean time to delivery following cervical ripening with dinoprostone.

	Mean time to delivery (hours and minutes)	P-value
All deliveries included	20h39 min ± 10h49 min	
Following dinoprostone + amniotomy + oxytocin	25h56 min ± 10h17 min	<0,0001
Following Dinoprostone alone	13h31 min ± 6h37 min	
Cesarean section	22h43 min ± 11h42 min	0,008
Vaginal delivery	19h40 min ± 10h18 min	
Parity		0,0014
Nulliparous	22h05 min ± 10h54 min	
≥1	18h36 min ± 10h23 min	
Age (years)		0,025
≤25	22h07 min ± 10h45 min	
26–30	18h35 min ± 10h37 min	
31–35	20h46 min ± 10h46 min	
≥36	22h52 min ± 10h55 min	
Gestational age (weeks)		0,729
<37	21h20 min ± 10h12 min	
≥37	20h38 min ± 10h53 min	
BMI (kg/m2)		0,017
<25	19h12 min ± 10h52 min	
≥25–29	21h35 min ± 10h27 min	
≥30	22h48 min ± 10h58 min	
BISHOP score		0,087
0–3	21h24 min ± 10h38 min	
>3	19h31 min ± 11h02 min	
Cervical dilation		0,041
Open	20h00 min ± 10h55 min	
Closed	22h30 min ± 10h22 min	
Premature rupture of membranes		<0,0001
Yes	17h48 min ± 10h00 min	
No	21h58 min ± 10h56 min	

Data expressed as mean ± standard deviation.

**Table 4:** Prediction model of time to delivery.

Model		Non-standardized Coefficients		Standardized Coefficients	t**	Significance	Confidence interval [95%]	
		B*	Standard error	Beta***			Inferior limit	Superior limit
1	Variable	961,188	134,997		7,120	,000	695,781	1226,595
	Parity	−80,346	30,030	−,133	−2,676	,008	−139,385	−21,307
	BMI	21,437	4,895	,215	4,379	,000	11,814	31,060
	Cervical dilation	−165,263	50,504	−,158	−3,272	,001	−264,554	−65,972
	Premature rupture of membranes	−241,759	67,246	−,172	−3,595	,000	−373,965	−109,552

*“B” quantifies the impact of each variable on the time to delivery. The + or − sign indicates the type of impact.

**“t” refers to the significance level of B.

***“Beta” refers to the change in standard deviation for the time to delivery for an increment of one standard deviation of the explanatory variable, when all other variables are constant.

## Discussion

To the best of our knowledge, our study is the first to develop a regression equation to predict the mean time to delivery following cervical ripening with intravaginal dinoprostone insert. The mean TTD was 20h39 min ± 10h49 min.

Wing *et al*. published a RCT in 2013 where they compared mean TTD following cervical ripening with intravaginal misoprostol and dinoprostone^8^. A total of 1358 women with a Bishop score ≤4 were randomized between cervical ripening with a 200 microgram misoprostol vaginal insert (n = 678) or a 10 mg dinoprostone vaginal insert (n = 680). The primary end points were time to vaginal delivery and rate of cesarean delivery. The median time to any delivery was 27.3 hours in the dinoprostone group compared to 19.2 hours in our study. Several factors could explain this important difference in the median time: the lower Bishop score (≤4 compared to ≤6 in our study), the higher rate of nulliparous (66% versus 59%), and the lower rate of cesarean section (27% versus 33%). It is worth noting that the cesarean section rate in nulliparous women following cervical ripening was 44% in our study, which is comparable to the 42% reported by Laughon *et al*.^22^.

We found several factors to be significantly associated with an increased time to delivery following cervical ripening with vaginal dinoprostone. The first was nulliparity, which has been reported as a risk factor for prolonged labor and time to delivery since the 1950s^23^. Several studies have indeed shown a significantly increased time to delivery in nulliparous compared to multiparous women^24–26^. In the study by Wing *et al*.^8^, the median time to vaginal delivery was significantly increased in nulliparous women (n = 451) compared to multiparous (n = 229) (43.1 hours vs 20.1 hours, p < 0.001, respectively), following cervical ripening with vaginal dinoprostone^8^.

The second factor was overweight, defined as BMI ≥ 25, a finding reported by Pevzner *et al*.^27^. Indeed, in a multicenter double-blind trial including 1273 women, they analyzed the impact of obesity on the outcome of cervical ripening with misoprostol or dinoprostone. The median time to delivery was significantly shorter in non-obese patients (22.7 hours) compared to patients with a BMI between 30 and 40 (24.9 hours) or > 40 (27 h) (p < 0.05)^27^. Another recent historical multicenter cohort study, which included 2122 IOL, out of which 1049 used misoprostol, 121 prostaglandins gel, and 74 a dinoprostone vaginal insert, while the rest were performed using only oxytocin or balloon catheters^28^. The induction to delivery interval was significantly shorter in patients with a BMI < 30 (25.8 hours) compared to BMI between 30 and 35 (27.8 hours), between 35 and 40 (29.1 hours) and BMI > 40 (31.7 hours). The authors concluded that an increased BMI had a negative impact on induction of labor at term^28^. To date, there is no clear explanation as to why the delivery interval is longer in obese patients, but one of the suggested hypotheses is a reduced uterine contractility in these women^27^. It would have been interesting to analyze the weight gain during pregnancy and its possible impact on the time to delivery for all BMI categories, but the data was not available.

The third factor we found to significantly influence the mean time to delivery was the initial cervical dilation, and not the Bishop score. A recent systematic review including 13 757 deliveries from 40 primary studies has also found that the Bishop score was a poor predictor for the outcome of induced labor at term and should therefore not be used to decide whether to induce women at term or not^29^. Moreover, Ducarme *et al*. found that the initial cervical dilation and consistency were correlated with failure of cervical ripening in post-term pregnancies^30^. Overall, initial cervical dilation seems to be a better predictive factor of time to delivery and outcome of induction than the Bishop score and could be the most important criterion when assessing women for cervical ripening and induction of labor. The fourth and last factor we found predictive of time to delivery was the state of the membranes–intact membranes associated with a longer time to delivery. This is in accordance with the recent prospective randomized trial by Bostanci *et al*.^31^. The study randomized 200 patients to two groups, following ripening with a 10 mg dinoprostone vaginal insert: early amniotomy (cervical dilation at 3 cm) or standard amniotomy (membranes left to rupture spontaneously). The median time from induction to delivery was significantly shorter for women who underwent early amniotomy (13.72 versus 22.73 hours, p < 0.05), and the frequency of vaginal delivery within 24 hours was significantly higher in this group (89% vs 45%, p < 0.05)^31^. In another RCT, Macones *et al*. compared TTD in 292 nulliparous women who had an amniotomy at ≤4 cm cervical dilation to 293 who had the amniotomy at ≥5 cm^32^. They also found a significantly reduced TTD (19.0 vs 21.3 hours) and a higher proportion of deliveries within 24 hours (68% vs 56%) in women with early amniotomy^32^. Overall, these results confirm that amniotomy decreases the time to delivery following cervical ripening. It is worth noting that amniotomy, in association with oxytocin infusion, is cited as a method of induction of labor in the guidelines of the HAS (Haute Autorité de Santé), ACOG (American College of Obstetricians and Gynecologists), and WHO (World Health Organization)^3,19,33^.

Based on the aforementioned factors, we have developed a mathematical model that would help predict the time to delivery following cervical ripening with dinoprostone vaginal insert. Indeed, this model integrates the four significant variables (parity, BMI, cervical dilation and rupture of membranes) with a coefficient taking into account the impact of each of these variables on the TTD. The regression equation we obtained allows therefore to calculate a personalized TTD for each patient, as follows:” Y = 961.188–80.346 × *parity* + 21.437 × *BMI*–165.263 × *cervical dilation*–241.759 × *PRM”*. The estimated TTD can be therefore calculated after the initial evaluation, and the admission and insert placement planned accordingly. This tool could be valuable for both obstetricians and patients. Indeed, it can help the organization in maternity wards, by scheduling vaginal insert placements according to the estimated TTD, and can help counseling women before admission to the maternity ward.

The main limitation of our study is the retrospective, single center design. Moreover, the mathematical model we developed was based on our patient population, and should therefore be validated in other populations with different demographics and obstetrical characteristics. It is worth mentioning that, even though the standard-deviation for the mean time to delivery for all patients is large, it does not negatively impact the power of our study. Indeed, we performed the multivariate analysis and the confidence intervals of explanatory variables for the mean time to delivery do not overlap zero. The analysis of residuals is centered on zero (cf supplementary information). The sign of the coefficient of each predictor remains the same regardless of the confidence interval: the direction of the relation between the explanatory and the response variable remains the same for each and every one.

The main strength of our study is the inclusion of a large and very well-defined population of women having cervical ripening with dinoprostone vaginal insert. Moreover, it is one of the few studies analyzing the different factors that could influence the time to delivery following ripening, and, to the best of our knowledge, the first to develop a mathematical model to help predict the time to delivery.

In conclusion, our study has shown that parity, BMI, initial cervical dilation and the status of fetal membranes significantly impact the time to delivery following cervical ripening with 10 mg dinoprostone vaginal insert. These factors were integrated into a regression equation in order to predict the time to delivery for each patient. We believe this equation could be an important tool to be included in organizational charts in busy maternity wards, and help schedule deliveries according to the risk factors and the ward’s activity. A prospective study is currently underway in order to validate our mathematical model (NCT 03482531).

## Supplementary information

Table of statistics residuals

## Data Availability

The dataset generated during the current study can be made available upon request to the corresponding author.
