# Supplementary material for: A mathematical model to predict mean time to delivery following cervical ripening with dinoprostone vaginal insert
Source: Sci Rep. 2019 Jul 9;9:9910. doi: 10.1038/s41598-019-46101-2 (PMC6616328; doi:10.1038/s41598-019-46101-2)
Supplement: Supplementary file 1 — Table of statistics residuals [file 41598_2019_46101_MOESM1_ESM.pdf]

A mathematical model to predict mean time to delivery following cervical ripening with dinoprostone vaginal insert.

Fanny Levast[1,*], Guillaume Legendre[1], Hady El Hachem[2], Patrick Saulnier[3], Philippe Descamps[1], Philippe Gillard[1], Pierre-Emmanuel Bouet[1]

[1] Department of Obstetrics and Gynecology, Angers University Hospital, Angers, France

[2] Department of Reproductive Medicine, Clemenceau Medical Center, Beirut, Lebanon

[3] Department of Methodology and Biostatistics, Angers University Hospital, Angers, France

[*]Correspondence and requests for materials should be adressed to F.L (email: fannylevast@yahoo.fr)

Table of statistics of residuals (a)

	Minimum	Maximum	Mean	Standard deviation	N
Predictors	710,5430	1893,6970	1236,7085	216,57145	398
Residuals	-1295,73462	2382,65210	,00000	616,30556	398
Standardized predictors	-2,430	3,034	,000	1,000	398
Standardized residuals	-2,092	3,846	,000	,995	398

“a” Dependent variable: Time to delivery
